# The role of de novo mutations in adult-onset neurodegenerative disorders

**DOI:** 10.1007/s00401-018-1939-3

**Published:** 2018-11-26

**Authors:** Gaël Nicolas, Joris A. Veltman

**Affiliations:** 1 Normandie Univ, UNIROUEN, Inserm U1245 and Rouen University Hospital, Department of Genetics and CNR-MAJ, Normandy Center for Genomic and Personalized Medicine, 22, Boulevard Gambetta, 76000, 76031 Rouen Cedex, France; 2 Department of Human Genetics, Radboud University Medical Center, Nijmegen, The Netherlands; 3 Institute of Genetic Medicine, Newcastle University, Newcastle upon Tyne, UK

**Keywords:** Alzheimer, Parkinson, Frontotemporal dementia, Somatic, Mutation, Mosaicism, De novo

## Abstract

The genetic underpinnings of the most common adult-onset neurodegenerative disorders (AOND) are complex in majority of the cases. In some families, however, the disease can be inherited in a Mendelian fashion as an autosomal-dominant trait. Next to that, patients carrying mutations in the same disease genes have been reported despite a negative family history. Although challenging to demonstrate due to the late onset of the disease in most cases, the occurrence of de novo mutations can explain this sporadic presentation, as demonstrated for severe neurodevelopmental disorders. Exome or genome sequencing of patient–parent trios allows a hypothesis-free study of the role of de novo mutations in AOND and the discovery of novel disease genes. Another hypothesis that may explain a proportion of sporadic AOND cases is the occurrence of a de novo mutation after the fertilization of the oocyte (post-zygotic mutation) or even as a late-somatic mutation, restricted to the brain. Such somatic mutation hypothesis, that can be tested with the use of novel sequencing technologies, is fully compatible with the seeding and spreading mechanisms of the pathological proteins identified in most of these disorders. We review here the current knowledge and future perspectives on de novo mutations in known and novel candidate genes identified in the most common AONDs such as Alzheimer’s disease, Parkinson’s disease, the frontotemporal lobar degeneration spectrum and Prion disorders. Also, we review the first lessons learned from recent genomic studies of control and diseased brains and the challenges which remain to be addressed.

**Electronic supplementary material:**

The online version of this article (10.1007/s00401-018-1939-3) contains supplementary material, which is available to authorized users.

## Introduction

The etiology of most of the adult-onset neurodegenerative disorders (AOND) is considered multifactorial, including genetic and environmental factors. In certain proportions of patients, the disease can, however, be inherited as a Mendelian trait, i.e., monogenic forms (Box 1). An autosomal-dominant pattern of inheritance is the most frequently encountered, so that family history is often positive for the same disorder. Such monogenic forms may be associated with extreme phenotypes and early ages at onset, but this is not always the case. The existence of patients with an extreme/early onset of AOND and a negative family history indicates that this disease is not always transmitted in an autosomal-dominant fashion. Autosomal recessive inheritance explaining the disease in some of these patients has been described, for example, in patients with Parkinson’s disease [62], but in some patients, causal mutations were observed in autosomal-dominant genes known to cause AOND [62, 83, 116, 168]. The primary hypothesis is that these mutations appeared in the germline of these probands as de novo mutations (DNMs). To prove this, however, analysis of parental DNAs is required. As the disease onset even in these extreme cases occurs relatively late in life, one of the main practical challenges is access to parental biological samples [148].

**Fig. 1:**
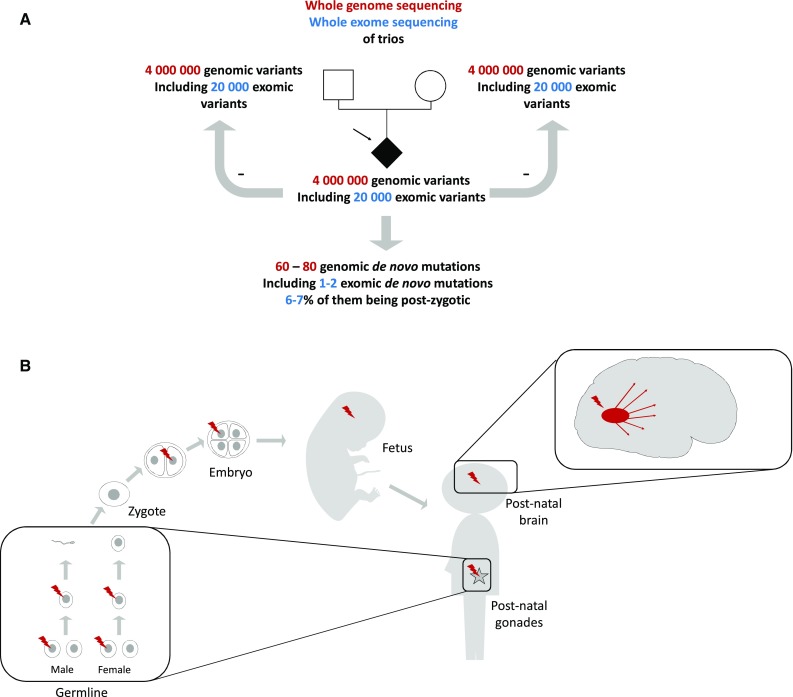
Germline, post-zygotic, and late-somatic de novo mutations: statistics, detection methods and their putative role in adult-onset neurodegenerative disorders. **a** Next-generation sequencing (NGS) consists of the massively parallel sequencing of short DNA fragments. This can be applied to the whole genome (WGS) or to targeted regions after the capture of regions of interest. For example, the capture of all coding regions allows the sequencing of the whole exome (WES). Whole genome sequencing (WGS) unveils around 4,000,000 variants (single nucleotide substitutions and short insertions and deletions) per individual genome. Among them, about 20,000 are located in the exons or canonical splice sites and hence detectable by WES. The trio study design consists of WGS or WES of a proband and his/her unaffected parents. After subtraction of all proband’s variants which were inherited from the parents, the variants that remain in the analysis are specific to the proband and are called de novo mutations (DNMs). WGS trio studies showed that about 60–80 high confidence DNMs can be identified per individual. Among them, 1.5 on average fall into the exome. **b** Among the DNMs identified per WGS of trios, about 7% have been shown to arise after the fertilization of the egg. These mutations are called post-zygotic or somatic mutations. During the entire life, every replicating cell can be affected by a novel mutation, giving rise to a colony of cells carrying this mutation. These mutations can arise lately in the development and during adult life, and possibly concern a single organ (late-somatic mutations). At most, a somatic mutation can concern a single cell. Although DNA replication is a main source of mutations in replicating cells, it has been shown that neurons, which are post-mitotic cells, can also be affected by novel, single cell mutations. These mutations are enriched in highly transcribed DNA regions, suggesting that neuronal activity can favor the occurrence of somatic mutations. DNMs can affect any genomic region. Hence, the nature of the biological consequence, if any, depends on the nature of both the region and the nucleotide change. While most of the DNMs do not have any significant biological consequence and do not cause any disease, some can result in a rare monogenic disease or modify the risk of developing a given disorder. In adult-onset neurodegenerative diseases, pathogenic DNMs have been mostly identified in known autosomal-dominant genes, such as *PSEN1* in Alzheimer’s disease, *FUS* in amyotrophic lateral sclerosis, or *PRNP* in Prion disorders. The most compelling evidence comes from germline DNMs, identified either by targeted genetic screening or by WES. In addition, WES studies revealed DNMs in novel candidate genes, but their rarity does not yet allow the measurement of their contribution to the disease etiology in these patients

The advent of massive parallel sequencing (also called next-generation sequencing, NGS) has allowed researchers to assess the de novo paradigm in sporadic AOND in a genome-wide manner using whole genome (WGS) or whole exome sequencing (WES). Sequencing of affected patients and their unaffected parents in a trio study design enables the identification of 1–2 DNMs per exome on average (for review see [3, [175] (Fig. 1a). This study design was originally applied to sporadic early-onset neurodevelopmental disorders and revealed a high genetic heterogeneity with many different genes affected by DNMs [42, 44, 69]. Although the application of the trio study design to sporadic AOND is limited by the access to parental biological samples, recent successful applications were published on Alzheimer’s disease (AD), Parkinson’s disease (PD), and amyotrophic lateral sclerosis (ALS) [37, 58, 84, 148, 162, 173].

Beyond germline mutations, there is now clear evidence that the human genome is mutable at any step of the development and during the entire life (Fig. 1b). In AOND, a putative role for somatic DNMs is currently being assessed [105, 112, 152]. The mechanisms of seeding and spreading of pathologic misfolded proteins are shared by multiple AONDs. It is thought to be a key mechanism explaining the irreversible progression of neurodegeneration throughout the brain [126]. In theory, a small amount of pathologic seeds synthesized by a “colony” of neurons carrying a given somatic variant could be a source of spreading of a pathological protein throughout the brain. The hypothesis that a putatively causal DNM could have happened after the fertilization of the egg (post-zygotic mutation) or even later during the development (late-somatic mutation) has been evoked quite early in the recent history of human genetics [183]. However, it remained difficult to assess until recently, because of technological limitations. Novel sequencing technologies now enable researchers to address this question accurately.

In this review, we report and discuss the existing evidence of de novo germline mutations identified in the most common AONDs as well as the increasing interest in the search for post-zygotic variants as candidate causal mechanisms in some patients with a sporadic presentation.

## Indirect arguments

The later a Mendelian disorder manifests in life, the more challenging it is to provide evidence of a de novo occurrence of causal mutations, because of reduced availability of parental samples. However, the late onset also has another effect: a significant difference between AOND and severe early-onset neurodevelopmental disorders—which have been shown to be largely caused by DNMs—is that AOND causing mutations do not affect the ability of the carriers to have children. Hence, pathogenic DNMs can be transmitted to the offspring. As with all genetic variants, mutations segregating in families arose de novo at a given date in a given individual. Hence, founder effects can be identified, and, eventually, it may be possible to date the original (de novo) mutation. A few of the recurrent mutations causing autosomal-dominant Alzheimer’s disease (AD) have been subjected to the study of a putative founder effect. The most famous one is certainly the Colombian *PSEN1* p.E280A mutation. Identity-by-descent analysis of the genomic sequence of 102 individuals originating from Antioquia provided the estimation of the DNM occurrence to 15 generations ago, back in the early 16th century [87]. Another well-known example is the Parkinson’s disease-associated p.G2019S *LRRK2* mutation, which is known to be present on different haplotypes suggesting different founders. One of them was estimated to have occurred 159 generations ago in a Berber founder [20]. The identification of recurrent mutations in autosomal-dominant genes in the absence of a founder effect or evidence for multiple founders points to the regular occurrence of DNMs in these genes. For example, the *PRNP* p.P102L and the p.D178N mutations have been reported in multiple pedigrees on different haplotypes and indeed recently DNMs have been identified at these positions [81, 182] [8, 41]. Likewise, the recurrent *SNCA* p.A53T mutation has been shown to have occurred on different haplotypes as well as genuine de novo events [77, 134, 136]. In addition to point mutations, other genetic variations such as copy number variations (CNVs, genomic deletions or duplications) may also occur de novo. The existence of shared breakpoints mapping to short tandem repeats on chromosome 21 among different families carrying diverse sizes of *APP* duplications indicates that multiple recurrent de novo duplications can cause monogenic forms of AD [149]. We can find similar evidence in synucleinopathies with *SNCA* copy gains (duplications and triplications) [147, 186].

## Methods and strategies for the detection of de novo mutations in AOND

De novo mutations may occur in any cell. When a DNM occurs in a parent’s germ cell, it is present in the fertilized oocyte and hence in every cell of the individual born from the development of this oocyte (Fig. 1b). Detecting a germline DNM is therefore relatively easy, requiring just a little bit of DNA from any tissue of the patient and his or her parents. Traditional and next-generation sequencing technologies allow the detection and validation of such mutation with a mutant: wild type allelic ratio of 1:1 (Table 1) in the patient, whereas the mutation should be absent in the DNA of both parents. In addition, it is important to check concordance of the patient–parent trios through segregation analysis to preclude false discovery of a DNM (e.g., false paternity). In AOND, the access to the parental samples remains an issue, limiting the detection of genuine DNMs. For these reasons, in a high proportion of the sporadic cases with a likely penetrant variation in a Mendelian gene, the evidence of the de novo occurrence could not be provided (e.g., [16, 85]).

**Table 1:** Main techniques for the detection of de novo mutations: pros and cons

	Germline DNM	Post-zygotic DNM
Sanger sequencing	Restricted to a few genes	Lack of sensitivity for mutations present in < 20% of cells
Targeted NGS (~ 100×)	Restricted to selected genes Can be analyzed affordably and at high throughput	Restricted to selected genes, but able to sequence many samples affordably Detection of mutations possible if present in > 10% cells
Standard WES (50–100×)	All coding regions, hypothesis free	Detection of mutations possible if present in > 10% cells
Standard WGS (15–30×)	All coding and non-coding regions, hypothesis free Better sensitivity for structural variations Relatively expensive	Detection of mutations possible if present in > 20% cells Relatively expensive
Deep NGS^a^	Not much added value compared to standard depth NGS	Increased sensitivity Cost increasing with the number of genomic regions sequenced Not affordable for WGS

*DNM* de novo mutation, *NGS* next-generation sequencing, *WES* whole exome sequencing, *WGS* whole genome sequencing

^a^Targeted, WES, or WGS. Note that the use of unique molecular identifier (UMI) may increase the accuracy of the variant calling for low-level mosaics by allowing the trimming of PCR duplicates and hence help distinguish true variants from PCR errors

The use of WGS or WES with a trio study design allows the detection of DNMs in a hypothesis-free way. DNMs can be detected in any gene, opening the way to the discovery of novel causal genes. Every individual genome contains around 4.1–5.0 million SNVs or indels as compared to the reference genome, of them, 20,000 on average map to the exons and can hence be detected by WES [3]. Of these 20,000 exonic or splice site variants, around 1000 are considered rare variants as they occur in less than 1% of the normal population. Sequencing a single exome or genome then faces the need to filter and prioritize these rare variants with the hypothesis that one of them could be causative. In sporadic diseases where the hypothesis that the cause could be a DNM, the bioinformatics subtraction of all variants identified in a proband that are also present in the parents (inherited variants) unveils the proband-specific variants, i.e., the DNMs [179]. On average, 40–80 DNMs can be identified per genome, of them 1–2 map to the exons ([3], Fig. 1a). This reduced number of candidate variants allows for a very effective follow-up in both research and diagnostics. For these reasons, trio-based patient–parent studies are now routinely carried out in medical genetics. Sequencing dozens of patient–parent trios may often turn out to be more powerful than sequencing hundreds of simplex cases of sporadic disease.

In clinical practice, the inclusion of a trio for a WES in the context of an AOND requires that (1) the parents are unaffected (2) there is no further family history in other generations, to reduce the risk of alternative mechanisms such as variants with reduced penetrance, (3) the parents are clinically accessible. This latter point is mandatory, both for checking the absence of disease and for DNA sampling after informed consent. In diseases with an onset after 50 years, it can be basically challenging to recruit unaffected parents with reduced mobility. Hence, only a small subset of patients and unaffected parents can be sampled, with a significant effort and organization being required to recruit each trio. However, one can hypothesize that the development of genomic medicine will be associated with a dramatic increase in the number of individuals undergoing WES or WGS during their lifetime, contributing to the success of future trio studies. Similarly, the development of large nationwide biobanks such as the UK biobank may in the near future also be very helpful for retrieving the genomic data of parents from tomorrow’s patients.

When a DNM occurs after the fertilization of the oocyte, this mutation is considered a post-zygotic DNM. When these post-zygotic DNMs occur early in the embryonic development, they may be present in majority of the individual’s cells, in tissues resulting from all three embryonic layers. Although post-zygotic and somatic mutations both refer to mutations being acquired during the lifespan of an individual [23], we will here use the term post-zygotic for mutations which have occurred during the early development, leading to their presence in most tissues from two or all three layers, and late-somatic mutations for those having been and being restricted to a single tissue, such as the brain. In addition, post-zygotic mutations may be transmitted to the offspring when present in the germline (germline or gonadal mosaicism) and explain how a germline heterozygous dominant de novo mutation may be present in affected sib pairs and appear to be absent in the parents. A combination of germline and somatic mosaicism (gonosomal mosaicism) can have clinical consequences or not in the carrier [23, 32].

WES and WGS technologies are based on the multiple sequencing of every base by multiple short reads in a paired-end sequencing manner, i.e., the sequencing of both extremities of a DNA fragment. The number of reads per position defines the depth of coverage. Most WES and WGS studies are performed with a depth of coverage allowing a high confidence in the detection of germline homozygous (~ 100% of alternate reads) and heterozygous (~ 50%) variants. When the alternate: reference allelic balance is different from these expected ratios, the existence of a post-zygotic DNM can be suspected [1]. However, technical artifacts are the main source of such altered allelic balances, reducing the accuracy in the detection of post-zygotic variants. For these reasons, specific bioinformatics tools and independent molecular confirmations are required to confirm the presence of a post-zygotic variant instead of a sequencing artifact or a germline DNM. It has been shown that 6.5–7.5% of the DNMs detected in blood by WES actually occurred post-zygotically [1, 96]. The lower the true allelic ratio is, the more difficult it is to identify it reliably. Increasing the sequencing depth and the use of unique molecule identifiers (UMI, allowing the trimming of PCR errors) help to increase the confidence in low fraction post-zygotic or late-somatic mutations (Table 1) [3]. The technical aspects and implications of neuronal post-zygotic mutations on aging have been recently reviewed [151, 177] as well as their putative role in neurodegenerative diseases [94]. The sequencing of DNA isolated from the diseased tissue may help to identify late-somatic DNMs. In AOND, the access to CNS tissue is highly limited in vivo. Studies focusing on late-somatic DNMs must therefore rely on autopsies of patients, including the input of brain banks. For multiple reasons, despite the development of such brain banks in many Western countries, the proportion of patients undergoing full autopsy remains very low, limiting the use of such facilities. In addition, it becomes clear that brain banks should store not only brain tissue but also other tissues, to allow a better characterization of post-zygotic events.

In addition to the detection of putatively causal post-zygotic mutations, the same technologies can also be used to search for (1) “back mutations” that reverse or partially correct a phenotype or (2) second-hit mutations that trigger the disease in the presence of an additional inherited germline mutation. Although examples of both mechanisms have been described in other diseases such as skin diseases [124, 125], cortical dysplasia [140] or the neurocutaneous disorder neurofibromatosis type 1 [157], we could not find any example in AOND. It could be that these mechanisms play a more prominent role in rapidly dividing cells. Of note, the existence of an inherited genetic variant protecting against Prion infection seems to be in line with this theoretical hypothesis [12]. In addition, we cannot exclude a two-hit mechanism in certain diseases. Of note, in the FTLD–ALS spectrum, several genes share common mechanisms of RNA metabolism perturbation [133]. One could expect that second-hit post-zygotic mutations in the same pathways may increase such pathogenic processes. This would even be consistent with the hypothesis of a multistep process for the development of ALS [5]. Likewise, in AD, any post-zygotic second mutation that would modify the production, aggregation, toxicity or clearance of the Aβ peptide could in theory influence the disease progression [33].

## De novo mutations in known autosomal-dominant genes

### Chromosomes and copy number variations

The major prevalence of Alzheimer’s disease and the presence of amyloid pathology observed very early in the life of patients with Down syndrome [55] suggested that an extra copy of the chromosome 21 might be sufficient to cause the disease. The identification of duplications of the *APP* locus (mapping to the chromosome 21) causing autosomal-dominant EOAD (early-onset Alzheimer’s disease, onset before 65 years) without Down syndrome [149] was of major importance to confirm the amyloid hypothesis stating that the Aβ peptide aggregation, produced following the processing of the APP protein, has a key role in AD pathophysiology. As most trisomy 21 cases occur de novo, it can be considered as the most common cause of DNM causing sporadic AD, although occurring in patients with a genomic disorder. The first de novo duplication of the *APP* locus, not encompassing the critical Down syndrome region, has been recently reported in a patient with neuropathologically confirmed sporadic EOAD [148].

The study of CNVs in AD has been performed with genome-wide screens by array CGH or SNP arrays in patients with or without a family history [65, 150]. For example, an array CGH screen performed in patients with EOAD identified a few candidate CNVs, some of which were identified in sporadic EOAD patients. Unfortunately, segregation of these CNVs could not be assessed in parents [150]. More recently, a de novo partial deletion of intron 1 of the *BACE2* gene was identified in a patient with sporadic EOAD [148]. It has been hypothesized that this deletion of highly conserved non-coding genic DNA could lead to decreased non-amyloidogenic processing of APP, but the functional consequences remain to be determined. In a WES study of 522 EOAD cases including familial and sporadic cases and 584 controls, a 17q21.31 duplication encompassing the *MAPT* gene encoding the Tau protein was identified in 4 probands [92]. In one of them, the duplication occurred de novo and the parents were indeed unaffected by any neurodegenerative disease. This observation, together with the segregation of the same duplication in one family with an EOAD clinical presentation and the absence in controls, was a strong argument suggesting causality. Interestingly, although the patients were selected for an accurate diagnosis of EOAD with clinico-biological arguments, this duplication may eventually cause a novel primary tauopathy, closely linked but neuropathologically distinct from AD.

Overall and despite the major role of CNVs in human genetics, only a few examples are known to cause autosomal-dominant AOND. Of them, duplications or triplications of the *SNCA* gene have been identified in families with Parkinson’s disease or Lewy body dementia. In three unrelated patients with Parkinson’s disease, increased dosage of the *SNCA* gene was identified as a DNM (Supplementary Table 4) [127, 128], present in 42–75% of oral mucosa cells of these patients, and absent or present at very low percentages in blood, indicating post-zygotic occurrence. However, given the complexity of distinguishing post-zygotic mutations from technical artifacts, it remains important to confirm these findings by independent technological approaches (see also below).

### De novo point mutations: an earlier age of onset is associated with an increased DNM detection rate

Genetic analysis of sporadic patients presenting either an early-onset AD or a late onset of AD (the most common form) has been performed since the identification of the first causative genes (e.g., [40, 155, 185] or even before, as a candidate gene [180], but most of them were negative. The first proven DNM in AD was a *PSEN1* pathogenic mutation identified in the blood of a patient with a disease onset at the age of 37 years, reported in the context of a screening of 13 sporadic AD patients with a very early onset (before 51 years) [48]. Later on, two novel cases of *PSEN1* DNMs were reported [56, 130] so that more than 10 years after the identification of the three known causal AOND genes *APP*, *PSEN1* and *PSEN2*, only three pathogenic DNMs had been published (Table 1).

**Fig. 2:**
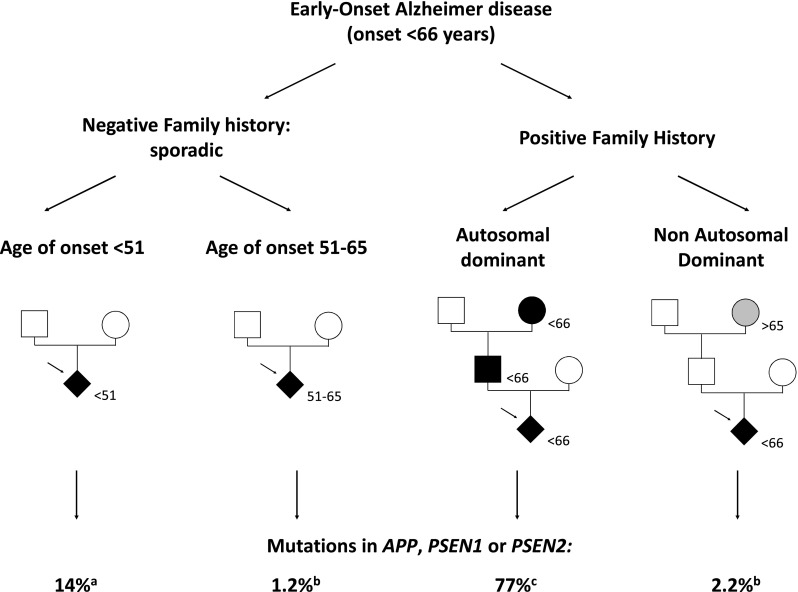
Mutation detection rates in autosomal-dominant genes: the example of early-onset Alzheimer disease. In sporadic patients with the earliest ages of onset, the mutation detection rate was higher than in patients with later ages of onset. This rate was mostly related to de novo mutations. When ages of onset are later, the proportion of inherited variants with reduced penetrance may increase. Note that the majority of these presentations may be non-Mendelian (complex determinism), whatever the age of onset. In familial presentations, the mutation detection rates were the highest when the family history suggested an autosomal-dominant transmission of EOAD (at least two generations with EOAD). In other cases (proband with EOAD, positive family history of Alzheimer disease with an onset after 65), the mutation detection rates are very low. In these forms too, a complex determinism is the most likely hypothesis. The mutation detection rates concern the three autosomal-dominant genes *APP*, *PSEN1*, and *PSEN2*. **a** Lanoiselee et al. [88] Plos Medicine 2017. **b** Nicolas et al. [116] European Journal of Human Genetics 2016. **c** Wallon et al. [181] Journal of Alzheimer’s Disease 2012

Recently, the genetic screen performed on blood samples of 129 sporadic patients with a very early age of onset (< 51 years) revealed a mutation detection rate of 14% (18/129) in the three known causal genes [88] (Fig. 2). Among them, the pedigrees of six showed a censoring effect (meaning that at least one parent died early, before the putative onset), parental DNA was not available although no censoring effect was noticed for nine, and parental DNA was available in ten cases, demonstrating the de novo occurrence of the mutation in each proband. Another screen performed by WES in 174 patients with sporadic EOAD starting between 51 and 65 years showed a much lower mutation detection rate of 1.2% in these genes (parental DNA not available, [116]). Taken together, this suggests that an earlier age of onset is a good predictor of a pathogenic variant in one of these genes and that such variants have a high probability to have occurred de novo (Fig. 2).

In addition to these ten aforementioned DNMs [88, 148], two additional *PSEN1* DNMs have been published [35, 99], as well as one post-zygotic *PSEN1* DNM [18] adding up to a total of only 14 DNMs ever reported as causing EOAD, all but one in *PSEN1* (Table 2, Supplementary Table 1). The only *PSEN1* post-zygotic DNM was identified in a woman with estimated degrees of mosaicism in blood and cerebral cortex of 8% and 14%, respectively [18]. She transmitted the mutation to her daughter, showing that the mutation encompassed gonadal cells. Notably, the mother presented a dementia onset at 52 years together with Parkinsonism and mild spastic paraparesis, whereas the affected daughter already presented the first symptoms of neurodegeneration at the age of 27, associated with dementia, cerebellar ataxia and spastic paraparesis. As this is a single family observation, it is unclear whether this difference in disease onset can be due to the germline vs. mosaic presence of the mutation (Table 2, Supplementary Table 1).

**Table 2:** Summary statistics of causal de novo mutations reported in known autosomal-dominant genes of the most frequent adult-onset neurodegenerative disorders

Nosological spectrum	Gene	Number of germline DNM	Number of post-zygotic DNM	Age of onset (average, range)	List of mutations and references
Alzheimer’s disease	*PSEN1*	13	1	35.9 [23–52]	Supplementary Table 1
	*APP* (duplication)	1	0	44	Supplementary Table 1
FTLD–ALS spectrum	*FUS*	18 (ALS)	0	22.3 [11–36]	Supplementary Table 2
	*MAPT*	4 (PSP,* n* = 1; bvFTD, * n* = 2; EOAD-like: * n* = 1)	0	39.25 [30–46]	Supplementary Table 2
	*SOD1*	1 (ALS)	0	20	Supplementary Table 2
	*VCP*	1 (ALS)	0	36	Supplementary Table 2
	*ERBB4*	1 (ALS)	0	45	Supplementary Table 2
Prion disorders	*PRNP*	5	1	28.25 [18–34]	Supplementary Table 3
Synucleinopathies (Parkinson’s disease)	*SNCA*	1	3^a^	26.5 [18–35]	Supplementary Table 4
Total		44	5	29.6 [11–52 ]	Supplementary Tables 1-4

*DNM* de novo mutation, *FTLD* frontotemporal lobar degeneration, *ALS* amyotrophic lateral sclerosis

^a^All are increased copy numbers

Similar to AD, the percentage of patients with pathogenic DNMs also seems to be highly correlated with the age at onset in other AONDs. For example, a frequency of 1% *FUS* mutation carriers was found among 500 cases with sporadic ALS (mean age at onset: 60 years), including one DNM in the blood of a patient with an onset at 36 years [167], but higher *FUS* mutation detection rates were reported when screening series of younger sporadic patients, as for example, 3/11 (27%) [188] and 6/14 (43%) [67] in patients with an age of onset of less than 25 and 35 years, respectively. The *FUS* gene is the most recurrently hit gene by DNMs in sporadic ALS with 9 different DNMs reported in 18 independent cases (Table 3 Supplementary Table 2) [4, 17, 31, 38, 39, 45, 66, 67, 78, 93, 167, 188], compared to only one in *SOD1* [7], one in *VCP* [13], one in the novel gene *ERRB4* [166] and two in the *MAPT* gene in the FTLD spectrum [25, 117], including one shared by two siblings indicating a mosaic DNM encompassing the germline in one parent [25].

**Table 3:** Summary of whole exome or whole genome sequencing studies of trios of the most frequent adult-onset neurodegenerative disorders

References	Disease	Inclusion criteria	Age at onset (mean, range)	No. of trios	Sequencing technology	Average non-synonymous DNM per proband	Analysis strategies and main results	Identification of novel candidate genes
Rovelet-Lecrux et al. 2015 [148]	Early-onset Alzheimer’s disease	Diagnosis supported by biomarkers or post-mortem examination, no family history of dementia in first and second degree relatives, parents alive with normal cognitive score	50 years (44–58)	14^a^	WES (Agilent SureSelect Human All Exon, Illumina)	1.00	CGH array (*n* = 14) followed by WES of trios (*n* = 12). Network enrichment analysis Abeta network): significant enrichment in DNM in the network compared to the rest of the exome	*VPS35, MARK4*. In silico and in vitro functional evidence
Kun-Rodrigues et al. 2015 [84]	Early-onset Parkinson’s disease	Age at onset < 40 years, typical presentation of PD with negative family history, absence of pathogenic mutations in any of the known PD genes	NA	21	WES (Nextera Rapid Capture, Illumina)	0.90	Protein network analysis (STRING).Follow-up analysis of the most significant genes by searching for a recurrence among > 1200 exomes of PD patients	*PTEN, VABP, ASNA1.* One additional *PTEN* variant but mis-segregation. Two additional cases with a *VAPB* variant without segregation data. No functional analyses
Guo et al. 2018 [58]	Early-onset Parkinson’s disease	Age at onset < 36 years, typical presentation of PD with negative family history, no genetic cause or environmental risk factor, no consanguinity	30.79 years	39^b^	WES (NimbleGen capture, Illumna)	0.89	Selection of 12 candidate genes among the DNM, replication in two case–control datasets (rare non-synonymous variants) Functional assessment in drosophila	One non-synonymous DNM in *NUS1* in a proband, enrichment in rare non-synonymous variants in the case–control analysis (OR = 11.13; *p* = 1.01 10^−5^), defects in climbing ability, reduction in dopaminergic neurons, in the brain dopamine levels in drosophila expressing siRNA against the fly NUS1 orthologue
Chesi et al. 2013 [37]	Amyotrophic lateral sclerosis	Sporadic ALS, no C9ORF72 repeat expansion	32.1 years (28.5–51.4)	47	WES (Agilent SureSelect Human All Exon, Illumina)	0.53	Functional gene annotation (DAVID) enrichment in the chromatin regulators category. Follow-up on the SS18L1 gene and search for recurrence among 62 exomes of familial ALS, identification of a novel missense variant	*SS108L1.* In vitro functional evidence
Steinberg et al. 2015 [162]	Amyotrophic lateral sclerosis	Sporadic ALS, no history of ALS, even if an ALS associated mutation was found in a known gene	46.1 years (26–59)	44^c^	WES (Roche NimbleGen SeqCap EZ Human Exome Library kits, Illumina)	0.39	Functional gene annotation meta-analysis with Chesi et al. (DAVID): enrichment in genes related to transcription regulation	*CHRM1?* Hit twice (Chesi et al. [37] + Steinberg et al. [162]) no functional analyses
Van Doormaal et al. 2017 [173]	Amyotrophic lateral sclerosis	Sporadic ALS, prescreening of *C9ORF72* ± *SOD1, FUS,* and *TARDBP*	NA	82^d^	WES (Roche NimbleGen SeqCap EZ Human Exome Library kits, Illumina, *n* = 61) or WGS (Complete Genomics, *n* = 21)	0.84	Functional gene annotation (DAVID), protein–protein interactions (DAPPLE), meta-analysis with Steinberg et al. [162] and Chesi et al. [37]: enrichment in phosphoproteins, no increased interaction	None

^a^Including one proband with a *PSEN1* DNM and one proband with an *APP* de novo duplication

^b^Including 20 quads (trios + one unaffected sib pair)—one trio was removed after quality assessment

^c^Including 4 probands with a pathogenic mutation in a known gene, inherited from an asymptomatic parent

^d^Including 1 proband with a pathogenic *C9ORF72* repeat expansion, inherited from an asymptomatic parent

Despite the difficulty to assess the de novo occurrence of a pathogenic mutation detected in a Mendelian gene in a patient with a sporadic AOND, we found a total of 49 published examples (Table 3, supplementary Tables 1–4). The vast majority of them was reported in recent years, with 18 out of the 29 DNMs (62%) hitting “old” genes (*APP*, *PSEN1*, *PSEN2*, *MAPT*, *SNCA*, *PRNP*, *SOD1*) having been reported in the 2010s. This highlights an increasing interest in sporadic AOND genetics, but it also indicates that novel sequencing approaches and the trio-based study design are successfully being used. The highest numbers of proven DNM have been identified in sporadic early-onset ALS, especially in the *FUS* gene (all with a disease onset before 40 years). ALS is associated with a short life expectancy following the diagnosis and young patients often present with highly penetrant variants. This may have facilitated the prioritization of pathogenic variants as well as the collection of parental samples for demonstrating de novo occurrence. Similarly, all patients with a proven DNM in a known Mendelian AD gene had a disease onset before 50 years (range: 23–47 years). This may lead us to think that DNMs mainly cause early-onset sporadic AOND. This observation, however, is impacted by the fact that it is almost impossible to assess the role of DNMs in late-onset patients, because parental DNA samples will mostly be unavailable. The low mutation detection rates of known AOND causative genes in older sporadic patients do, however, suggest that this is not the only factor. It is well-known that early-onset severe disorders are more often monogenic and de novo in origin, whereas late-onset diseases show more complex inheritance [175].

### Recurrence of DNMs

Among the AOND genes affected by DNMs, *PSEN1* (AD), *FUS* (ALS) and *PRNP* (Prion disorders) are mostly reported (Table 2). Of the 11 missense *PSEN1* DNMs reported, 8 mapped to genomic positions already identified by another or the same causative mutation in families, and two additional ones mapped to a same codon. Three DNMs in FUS were identified as recurrent DNMs: one missense (c.1574C > T, p.Pro525Leu in 8 patients) and two protein-truncating mutations (one nonsense and one frameshift indel, each observed twice as DNMs). Interestingly, four additional *FUS* DNMs affected genomic positions where the same change had been reported as pathogenic in sporadic cases or families. Although no parental data was available for those cases, it still does indicate that these positions are affected by recurrent DNMs. In addition, two *FUS* DNMs are also present in the gnomAD database consisting of genetic variations identified in controls free of severe pediatric disease, with a maximum allele count of 5 (out of ~ 138,000 individuals) [95]. One of these was the recurrent nonsense DNM. This suggests that, although cases with a DNM in *FUS* were all young, the mutations may not be fully penetrant in other genomic contexts. Of the six reported *PRNP* DNMs, including one possibly post-zygotic mutation [8], all were reported in additional independent cases or families [34, 41, 63, 81, 154]. This may suggest the existence of hotspots or recurrent mechanisms such as octapeptide repeat expansions in the Prion gene.

Interestingly, no *APP* or *PSEN2* SNV has ever been reported as occurring de novo in a patient with AD. There is no evidence of a higher mutability of the *PSEN1* gene as a whole [95, 153]. However, *PSEN1* pathogenic variants are more frequent in autosomal-dominant families and *PSEN1* mutation carriers exhibit an earlier age at onset on average, compared to the other genes [88, 181]. This may help physicians to get access to parental samples at the time of their child’s diagnosis and introduce a bias in this analysis.

### Repeat expansions

There are multiple examples of neurological disorders caused by repeat expansions. Intra-familial instability of such repeats has been identified in diseases such as Friedreich’s ataxia, DRPLA or Huntington’s disease (for review, see [129]). Such instability is a particular form of a DNM that can lead to anticipation of the age of onset with a few dramatic examples [61]. In the main AOND, GGGGCC repeat expansions in the *C9ORF72* gene are also subject both to intra-familial and somatic instability [53, 164]. However, the ranges of repeat sizes conferring instability that can be translated to a pathogenic expansion in the offspring or in other cells of the body remain to be identified. By analyzing blood and CNS samples of patients with ALS, it has been suggested that such instability is not likely to be of sufficient magnitude to result in a pathogenic expansion in the CNS from a normal *C9ORF72* repeat length in blood [120]. Similarly, *ATXN2* intermediate repeat expansions have been shown to increase the risk of FTLD–ALS and have been reported as somatically instable [86, 111, 174].

### Monogenic causes in sporadic patients: alternative hypotheses

Beyond the possibility of a DNM hitting a gene with an autosomal-dominant pattern of inheritance, several alternative hypotheses remain consistent with a monogenic cause for sporadic AOND patients. Most obviously, several forms of sporadic early-onset Parkinson’s disease (PD) cases are related to autosomal recessive inheritance—with early and possibly clinically distinct clinical presentation—as is the case for a few examples in FTLD–ALS or related, sometimes clinically undistinguishable disorders [22]. In addition, in PD, the identification of an inherited pathogenic mutation in a dominant gene in a sporadic case is not uncommon [83]. Although some of the presentations are clinically distinct including an early onset, reduced penetrance has been identified for a subset of the genes and pathogenic mutations. We could find only one detailed report of a likely DNM in a known autosomal-dominant PD gene [136] (Supplementary Table 4). Hence, sporadic presentations may be more frequently due to autosomal recessive inheritance or autosomal-dominant inherited mutations with reduced penetrance rather than DNM in PD, aside from complex forms. Although autosomal recessive inheritance is not uncommon for known Parkinson’s disease genes, it remains unclear if this will also be the case for the remainder of causal genes to be discovered. A recently published WES study indicated that this may not be the case [71]. Similarly, the pathogenic *C9ORF72* repeat expansion causing FTLD–ALS was reported in 5% of sporadic cases [168] and may be much more frequently inherited from an asymptomatic parent than occurring de novo [108]. In AD, a few mutations have also been reported as associated with reduced penetrance (e.g., *APP* p.A713T, *PSEN1* p.A79V [88]) and only one mutation in *APP* is considered causing AD in a homozygous state [47].

Of note, in addition to autosomal-dominant variants with reduced penetrance and autosomal recessive inheritance, sporadic occurrence might also be related to di-, oligogenic or a more complex inheritance. Some AOND such as AD and ALS exhibit a high heritability as measured by comparing concordant and discordant monozygotic and dizygotic twin pairs (58–79% depending on the models for AD [52] and 38–74% in ALS [6]), whereas this was more modest in Parkinson’s disease (34%) [184]. In FTLD, it has been estimated that in most of the cases, frontotemporal dementia would be a genetic-based disease, even late-onset cases [26]. The inheritance patterns may be more complex in some instances, as suggested by the observation of damaging *TBK1* as well as *OPTN* mutations in a patient with FTLD-TDP [132]. In AD, the high odds ratios of risk factors such as the common APOE4-4 genotype or rare variants in *TREM2*, *SORL1* or *ABCA7* in EOAD (for review, see [113]) suggest that they contribute significantly to the genetic component of EOAD in a complex determinism, including sporadic and familial presentations. Likewise, in Parkinson disease, high odds ratios associated to heterozygous *GBA* variants suggested oligogenic inheritance in some cases [100]. Although a complex etiology might be relevant for a certain proportion of patients with sporadic AOND, it remains difficult to predict which patients, based on clinical criteria.

## Looking for novel genes hit by de novo mutations: whole exome sequencing of trios

The evidence that highly penetrant mutations can arise de novo in known Mendelian genes was a proof of concept allowing the assessment of the de novo paradigm at the scale of the entire exome, looking for novel genes. As for the known autosomal-dominant genes, applying the trio study design requires the access to parental biological samples. To date, six trio studies have been published: one in AD, three in ALS, and two in PD, with a total of 247 trios included (Table 3).

### Sporadic early-onset AD: enrichment of DNM in the Aβ network and in vitro characterization

After blood sampling of 14 patients with EOAD (mean age at onset: 50 years) and their unaffected parents and CNV screen by array CGH, WES of the 12 trios with no de novo CNV revealed 12 de novo non-synonymous variants in 6 patients, including one of the above-mentioned *PSEN1* DNMs [148] (Table 3). A significant enrichment of non-synonymous DNMs was found in a network of genes centered on the Aβ peptide. Of particular interest, two novel genes affected by DNMs were further studied: *VPS35* and *MARK4.* In silico and in vitro studies showed a deleterious effect of these DNMs with functional results being totally in line with AD pathophysiology. The *VPS35* missense DNM was shown to result in reduced protein function and hence predicted to result in increased Aβ secretion. This mutation had striking different functional consequences than the previously reported Parkinson disease-causing *VPS35* variant, even though both affected amino acids are located close to each other in the protein sequence [148, 178, 187]. The *MARK4* variant increased the phosphorylation of Tau in a key position for Aβ-mediated toxicity. The trio strategy pointed here two novel genes, not previously related to AD genetics but whose gene products are key players in AD pathophysiology. One can hypothesize that they contributed significantly to the development of the disease in their carriers, but their penetrance cannot be assessed without segregation data in the offspring of the carriers or recurrence in unrelated individuals, currently limiting the impact for genetic counseling.

### Two independent studies in ALS reveal one recurrently hit gene and pathway enrichment

In sporadic ALS, three trio studies using WES performed from blood samples were published. A first study reported 47 trios (mean age at onset: 32.1 years) and identified 25 genes hit by non-synonymous DNMs [37] (Table 3). After functional annotation, a significant enrichment in genes encoding chromatin regulators was found (5/25), including the neuronal chromatin remodeling complex component *SS18L1* (also known as *CREST*). In a replication sample of 62 WES of families with ALS, a novel missense *SS18L1* mutation was found in a proband. Both mutations inhibited activity-dependent neurite outgrowth in primary neurons, and CREST protein was associated with FUS in neurons. In an independent screen of *SS18L1* in 87 patients with familial ALS, two novel mutations were identified in unrelated probands [169], although one latter patient also presented a potential pathogenic mutation in the *OPTN* gene (a gene associated with recessive or dominant FTLD–ALS). Although the role of *SS18L1* and other genes hit by DNMs in this first WES study remains to determined, this study design unveiled good candidate genes to be studied further.

The second study enrolled 44 trios for WES (mean age at onset: 46.1 years) including one patient with FTLD–ALS (Table 3) [162]. Importantly, patients carrying pathogenic mutations in known genes were not excluded, as two carried a *C9ORF72* repeat expansion, one a *SOD1* mutation and one a *TARDBP* mutation; these mutations were inherited from an asymptomatic parent. Of 54 DNMs identified, 17 non-synonymous or canonical splice site mutations were found in 12 trios (or 15 mutations in 11 trios after excluding a trio carrying a *C9ORF72* repeat expansion with two non-synonymous mutations, [162]). Taking into account both WES trio studies together, one gene, *CHRM1*, was hit more than once, by a novel missense [37] and a start loss DNM [162]. While very exciting, the interpretation of the role of *CHRM1* DNMs in ALS pathophysiology remains to be assessed by functional analyses. In a similar in silico functional enrichment analysis using the DAVID bioinformatics tool as in the first study, the genes related to transcription regulation were highlighted as enriched in DNMs [162]. These first two trio studies performed on sporadic ALS highlighted the genetic heterogeneity and the need to analyze multiple trios at the same time or in the context of meta-analyses.

Recently, 82 additional sporadic ALS trios were reported (WES, *n* = 61; WGS, *n* = 21) [173]. No additional recurrence was identified at the gene level. The interpretation of results was conflicting with previous reports as global analysis of all genes hit by non-synonymous DNMs among the 173 published trios did not reveal any enrichment in the previously identified categories of genes (chromatin regulators, transcription regulation). Instead, an enrichment in the phosphoproteins category was identified, and no increased protein–protein interactions were identified as compared to the known interactions among known genes.

### Protein–protein interaction and recurrence of variants in large cohorts of Parkinson disease patients

One WES study was performed on 21 trios of early-onset PD (onset before 40 years) [84] (Table 3). Twenty DNMs (19 non-synonymous) were identified for which in silico functional gene annotation and protein–protein interaction analyses were performed using the STRING bioinformatics tool. Three genes showed significant interactions with the known PD genes: *PTEN*, *VABP*, and *ASNA1*. Additional rare variants were identified from exome data of more than 1200 cases from the International Parkinson’s Disease Genomics Consortium (IPDGC). Although functional pathways link *PTEN* to PD pathophysiology, the putative role of the DNMs found in patients with PD remains to be elucidated. In particular, the DNM identified in the replication sample is predicted to result in a loss of function and hence supposed to cause Cowden syndrome. In addition, while this variant occurred de novo, the proband’s father was also affected with PD, showing non-co-segregation. *VAPB* missense mutations have been previously shown to cause autosomal-dominant ALS. The patient identified in the PD trio study presented an unknown in-frame deletion of one residue and two additional cases from the IPDGC dataset presented rare coding mutations in the same domain as the one reported to carry ALS-causative mutations. Whether or not these mutations have a functional effect and are related to PD pathophysiology remains to be determined.

Very recently, a trio-based WES study reported 39 early-onset PD patients of Han Chinese origin [58]. After prioritization of DNMs based on protein–protein interactions and expression profiles in the brain, 12 genes were selected for case–control sequencing analyses. Among them, *NUS1,* which showed a splicing DNM in a proband, was enriched in non-synonymous rare variants with an OR of 11.3 and a *p* value approaching exome-wide significance (1.02 10^−5^) among 5089 cases and 4423 controls. In addition, a Drosophila model expressing an siRNA against the fly *NUS1* orthologue showed decreased climbing ability, a reduction in dopaminergic neurons and a dramatic reduction in the brain dopamine levels. Of note, all coding variants identified in the replication case–control analysis were missense and their inheritance was unknown, but the DNM mutation introduced a splicing defect resulting in the frameshift deletion of 91 bp of exon 3. This could suggest a haploinsufficient mechanism. However, protein-truncating *NUS1* DNMs have previously been reported in patients with global developmental delay and seizures [60], two of them also had a tremor. In addition, a 6q22.1 microdeletion, the critical region of which encompassed this gene, has been identified in patients with early-onset seizures [165] and a homozygous missense variant resulting in a protein loss of function was reported in two siblings with a congenital glycosylation disorder [123]. Given the very early onset of neurological signs in the *NUS1* DNM carrier in the PD study (16 years), the in-depth phenotypic description of patients carrying DNM will be of interest to better characterize the clinical expression of these mutations, in addition to the replication in other WES studies.

### WES of trios in AOND: validation warranted

We found six trio studies applied to sporadic AOND (Table 3), all with limited samples sizes: one in AD (14 trios), three in ALS (total of 173 trios), and two in PD (total of 60 trios). Yet, the power of these strategies allowed the identification of at least two novel genes in EOAD genetics with a demonstrated functional effect (*VPS35* and *MARK4*) [148], one in ALS (*SS18L1*) [37], and one recently in PD (*NUS1*) [58]. Other variants, affecting genes not yet known to be involved in the Aβ network in AD, or the recurrently hit gene *CHRM1* in ALS, might also be confirmed as truly involved in further studies.

Although the extreme resolution of the trio study design makes it a choice strategy in extreme presentations of sporadic AOND with no pathogenic variant in known genes, the identification of a DNM in a patient with a sporadic AOND is not sufficient to confirm its role in the disease etiology. Validations are mandatory to confirm the involvement of a novel gene identification through this process. Several strategies can be proposed: (1) functional assessment of variants, (2) recurrence in independent cases, (3) variant enrichment in case–control studies.

1. Functional assessment of the impact of DNMs relies on the previous knowledge on the disease pathophysiology and mutated genes. It was possible to assess the role of *VPS35* and *MARK4* mutations mostly because the function of their gene products was well-documented and because AD pathophysiological pathways, although diverse, can all be linked to the amyloid cascade hypothesis [33]. Regarding the *SS18L1* gene, the known expression in motor neurons was a strong argument to justify its assessment, although the effects of the observed mutations might not be specific to ALS pathophysiology. In PD, although a direct functional link between *NUS1* and PD was not known, a use of a clear biological read out in the Drosophila model (dopaminergic impairment) pointed to the relevance of this gene [58]. It is, however, very challenging to perform a systematic functional assessment on all DNMs identified in a WES trio study and therefore in most cases only strong candidates are selected for functional validation.
2. The argument of recurrence, at the variant or at the gene level, in patients with the same phenotype, is a key argument in human genetics. A good example of this is the observation of recurrent DNMs in the *CHRM1* gene in ALS by the combination of the first two studies. However, it is important to assess the a priori probability of a gene to be hit by multiple DNMs by chance, because the DNM rate varies per gene, depending on characteristics such as the gene size and the GC content [153]. The statistical rigor required to demonstrate a significant enrichment of DNMs in a gene may require the analysis of many hundreds to even thousands of trios [43], which seems unrealistic in AOND. As discussed before, however, most of the AONDs are compatible with parenthood. As for *PSEN1*, *APP*, or *FUS* mutations, for example, one could expect to find pathogenic mutations in novel genes such as *VPS35*, *MARK4*, *SS18L1*, *CHRM1* segregating in families if these mutations are highly penetrant. It may therefore be useful to perform replication studies in large cohorts, whether or not the patients present a positive or a negative family history, as performed in PD and ALS studies [37, 58, 84]. However, without sufficient segregation data as in the above-mentioned examples, no conclusion can be drawn.
3. The recurrence of rare variants at the gene level can also be assessed by association analysis in a case–control setting, as successfully applied for *NUS1* rare variants in PD [58]. WES or WGS data are rapidly increasing in terms of sample sizes, allowing for powerful association studies of rare variants. For example, about 5000 late-onset AD cases and 5000 controls have been exome sequenced by the US governmental AD Sequencing project (ADSP, [24]) and WES of 1779 AD cases and 1273 controls have been included in association studies in France [19]. So far, genes affected by DNMs in trio studies have not been reported to be significantly associated with AD in these association studies. Notably, the major roles of *TREM2*, *SORL1* and *ABCA7* rare variants have been highlighted in AD [19, 73, 91, 114, 163]. As expected, an apparent inverse correlation was identified between the effect size at the gene level (measured by the odds ratios, OR) and the cumulative frequency of the variants in controls: the genes with the highest OR (*TREM2 *> *SORL1 *> *ABCA7*) were hit by damaging variants in the inverse order in terms of cumulative frequency [19]. This observation, added to the full penetrance of autosomal-dominant pathogenic variants that are extremely rare in controls, is compatible with the paradigm reported by Manolio et al. [101] and McCarthy et al. [102] suggesting such inverse correlation for most of the association signals which can be detected using current methods. If DNMs identified in trio-based studies eventually carry a strong effect, they are likely to be extremely rare, so that association studies might fail in reaching the 2.5 10^−6^*p* value threshold required for exome-wide significance. Conversely, their extreme rarity is not sufficient to postulate on penetrance. The trio study design having an individual resolution, it remains possible that mutations in novel genes might be present only in a very limited number of cases in the world, precluding any confirmation by identifying recurrences leading to a significant association signal.

Lacking gene level evidence for most of the genes, WES/WGS trio studies attempted to find enrichment of DNMs at a larger scale by grouping genes into networks or pathways. Although this approach adds evidence to the study model, it does not allow the investigators to draw conclusions about the role of individual genes. Even in cases of a demonstrated functional effect, no genetic counseling can be provided to the carriers’ offspring and to putative unrelated carriers of rare variants in these genes.

Overall, when large series of patients with sporadic presentations of extreme phenotypes of AOND were screened for known Mendelian genes and by WES, a large proportion remained genetically unexplained. The application of WGS may further expand the spectrum of causal DNMs by allowing for a better coverage of the exome, the identification of non-coding DNMs as well as balanced and unbalanced structural variants [54]. In addition, the recent development of more sensitive and specific next-generation sequencing applications has allowed researchers to test an alternative hypothesis: highly penetrant post-zygotic DNMs might explain part of these sporadic presentations.

## Somatic mutations in adult-onset neurodegenerative disorders

The hypothesis that a genetic mutation present in only a proportion of the neuronal cells can cause a neurological disease has been formulated quite early in the history of AOND genetics [139, 180]. To date, most studies still focus on the analysis of germline mutations present in all cells by studying DNA isolated from a large proportion of blood cells. However, recent improvements in sequencing technologies have enabled the accurate identification of post-zygotic including late-somatic mutations present in subsets of cells or even in single cells [3]. In different neurodevelopmental disorders, evidence has been provided that post-zygotic or late-somatic mutations can cause disease using a combination of technologies on different tissues [70, 141]. Some of these mutations were detected in blood samples, indicating that they occurred early during development. One can assume that post-zygotic mutations, if detected in multiple tissues or with high allelic ratios in blood, might be present in a significant proportion of brain cells. Hence, the level of causality between germline and post-zygotic functional variants should be comparable. It is much more complex to detect brain-specific somatic mutations. Autopsy of certain AOND cases sometimes reveal widespread neuropathological lesions, which would be more in line with germline causes of disease. A focal onset of disease, as seen in some types of primary progressive aphasia in the FTLD spectrum, on the other hand, suggests a role for late-somatic mutations. Overall, in neurodegenerative disorders eventually affecting a large part of the brain, one could assume that the causative mutations must be present in a high proportion of brain cells (neurons and/or glial cells). However, most of the AOND share mechanisms called seeding and spreading. These features, also referred to as Prion-like properties, are conferred by proteins that can transfer their pathogenic state into wild type, normally folded proteins (seeding) and then spread into the whole brain following neuronal connections (for review, see [126]. This phenomenon has been studied first for the Prion protein itself. However, such properties are now being characterized for Tau, Aβ, TDP-43, α-synuclein, or even the Huntingtin protein even if they are not associated with a spontaneous infectious propensity. Similar to an external focal injection of pathologic proteins in animal models, one can hypothesize that a small proportion of cells carrying a somatic variant resulting in the production of a pathogenic misfolded protein could be the source of a cerebral neurodegenerative disorder. Low-level mosaics should therefore also be considered.

### Lessons from control brains and clues for the interpretation of somatic mutations in AOND

Recently, novel sequencing approaches provided critical knowledge on post-zygotic variation in healthy control tissues. The human genome is clearly not stable throughout life and post-zygotic variants may occur in any cell at any time (Fig. 1b). Replicating cells are particularly prone to somatic mutations, with highly replicating tissues such as the skin or hematopoietic tissue showing the highest somatic mutation burdens. External factors may favor the occurrence of mutations during the replication phase of the DNA, including mutagenic agents such as radiation or toxic agents. Aging seems to be the strongest risk factor influencing the accumulation of somatic mutations during clonal hematopoiesis [2]. We summarize hereafter the main points that we consider of high importance for the analysis and interpretation of somatic variants, following the study of normal and diseased brains.

1. Post-mitotic neurons exhibit an unexpectedly high burden of post-zygotic mutations. The use of single-cell genomic approaches including WGS in neurons from non-diseased brains unveiled an unsuspected amount of post-zygotic mutations, including about 1500 somatic SNV per neuron [98]. Of them, some occurred during fetal development [15, 75], but a higher burden was detected in post-mortem adults [98]. Although the number of single neurons that have been sequenced remains limited (dozens or hundreds), the elevated rate of post-zygotic SNV was quite unexpected as neurons are post-mitotic lifelong cells and hence are not subjected to errors during DNA replication, beyond the ones putatively acquired during the divisions of progenitors. Single neuron somatic SNV were mostly associated with transcriptional activity, i.e., neuronal activity, contrary to the variants shared by multiple cells in the brain or tissues with a high replication rate [98]. In addition, neurons may accumulate late-somatic mutations during aging [97]. Whatever the associated mechanisms, some of these events could result in the production of an abnormal/misfolded protein, which could represent a source of seeding and spreading in the brain, causing a neurodegenerative disease.
2. Bulk brain tissue is a combination of replicating and non-replicating, post-mitotic cells; sequencing genomic DNA isolated from bulk brain tissue does not allow the distinction between the cell types [64]. The identification of very low-level mosaics from bulk brain tissue does not imply that different cell types carry the mutation of interest and that the mutated genes are expressed in the mutated cells, hence leading to the production of an abnormal protein.
3. Although the access to brain tissue is mandatory to assess the somatic variant hypothesis thoroughly, studying other tissues may be worth of interest. It has been shown that mutations present in more than 5–10% of the brain cells were generally also detected outside the brain, in tissues derived from all three embryonic layers, including the ectoderm, suggesting that these mutations occurred during early phases of embryonic development [98]. Although this still requires replication, this is a strong argument for assessing other tissues, including ectodermal tissues and blood, when cerebral tissue is not available. The fact that some neuronal cells shared more common cellular ancestors with cells from other organs than the brain in one individual was also a surprising but promising finding for deep-sequencing studies performed on other tissues than the brain in AOND. However, every study performed from non-CNS tissue will be facing the non-representativeness of the allelic ratios eventually identified, and the lack of evidence that a putatively causal mutation with a low allelic ratio is really present in the affected neuronal cells. Importantly, recent results of single neuron WGS also raised the question of thresholds: if a putatively pathogenic mutation is found in a brain with an AOND, how many neurons should carry it, among the tens of billions of neurons in the brain [14], to be significant enough to cause a widespread neurodegenerative disease?
4. Data from mouse models suggest that seeds from peripheral tissues like blood [28] or intestine [82] can spread into the brain and cause a neurodegenerative disorder. The presence of a pathogenic mutation in the brain cells would hence not be mandatory to cause such a disease, although one can assume that a significant amount of pathological seeds should be produced to trigger an AOND.
5. The study of somatic aneuploidy and CNVs still requires technological improvements. Aneuploidy has been studied in AD brains for decades (for review see [9, 131, 145, 146], mainly thanks to slice-based cytometry and fluorescent in situ hybridization (e.g., [10, 106, 146]). The fraction of neurons containing extra chromosomes has been reported to be higher in AD brains than in controls [106]. Controversial results on aneuploidy rates ranging from 1% or less to more than 50% percent have been reported, as recently reviewed together with other inconsistencies (see [145, 146]). After the introduction of single cell NGS, the fraction of aneuploid neurons has been reevaluated to be from 0 to 3% [30, 80, 103, 172] and the rate of neurons carrying post-zygotic CNVs has been evaluated to several dozen percents. Given the challenging assessment of germline CNVs using NGS in general, the interpretation of CNVs from single cell WGS may also require caution and improved techniques are required as proposed recently [144]. In addition, it has been shown that DNA isolation protocols may significantly influence CNV detection [109]. Similar to CNVs, a high burden of post-zygotic L1 insertions that can disrupt or deregulate the expression of genes has been reported, although there are differences of opinions on the numbers of post-zygotic L1 insertions in single neurons [50, 171]; some results may require technical validation.
6. The study of brains from AOND cases implies the analysis of patients with advanced disease, which can be associated with secondary DNA damage. Increased aneuploidy rates and/or of DNA content in AD neuronal cells could be related to errors during mitosis of neuronal progenitors. More likely, though, it can be caused by a reentry in the cell cycle as the result of AD pathophysiological processes. This has been suggested by the identification of neurons with 4n DNA content and a positive staining for Cyclin B1 [106]. It is likely that, in the context of advanced neurotoxicity, such observation could basically be a simple consequence of neurodegeneration—nonspecific to a given AOND instead of a causative mechanism [9]. In theory, the study of so-called preclinical AD brains could help tackle this issue. However, a proportion of neurons in preclinical AD brains might already be at a final stage of the pathophysiology. Studies of presymptomatic patients carrying *APP*, *PSEN1*, or *PSEN2* mutations showed evidence of neuronal damages years before the first clinical signs, similar to what has been observed in animal models [21, 79, 131]. Even if a few studies had access to samples of patients with preclinical or early-stage AD, most research is performed on end-stage AD as it has been the main condition leading to the patient’s death. While many neurons already died, many other neurons have undergone stress and toxicity during years, before the first symptoms appeared. Some of them have accumulated DNA damage, including somatic SNVs [97]. Oxidative stress and microtubule dysfunction in AD neurons are several of the causes leading to secondary damages in the DNA. In FTLD and ALS, it has been shown that secondary DNA damage can participate in the pathophysiological processes in *C9ORF72* and *FUS* mutation carriers [51, 110]. Interpreting genetic results from AOND brains should therefore be done with great caution. Of note, similar caution should be provided for the interpretation of somatic mitochondrial DNA mutations which face the same issue of possible secondary mutations induced by neurodegeneration. In addition, caution is required when interpreting negative findings in sequencing studies performed on brain tissue. Indeed, the mutated cells may also be the more vulnerable ones and hence mutations may be undetectable because of cellular death.

Taken together, somatic mutations in the brain may result from (1) early embryonic events, (2) mutations occurring in neuronal progenitors during neurodevelopment as a result of replication errors, (3) mutations in replicating cells in the brain at any stage of life as a result of replication errors, (4) mutations in post-mitotic neurons as a result of transcriptional activity and (5) as a result of DNA damage in the context of cellular stress. Although advances in genomic and single cell technologies have provided novel information and promising hypotheses, the interpretation of sequencing data obtained from brains with AOND will be an even bigger challenge than the technology itself in the near future.

### Somatic mutations in patients with AOND

Given the role of germline duplications of *APP* and *SNCA*, respectively, in autosomal-dominant EOAD and Parkinson’s disease, CNV studies have focused on these loci as well as chromosomal abnormalities. There is still debate on whether AD brains are enriched in neurons carrying extra copies of chromosome 21 containing the *APP* gene. Interestingly, Bushman et al. [29] recently reported increased copy numbers of the *APP* locus itself in AD brains. So far, however, this exciting result has not been replicated. Even more recently, the study of nigral dopaminergic neurons—the neurodegeneration of which causes Parkinson’s disease—revealed an average proportion of dopaminergic neurons with gains of *SNCA* copies in each nigra of 0.78% in Parkinson disease patients versus 0.45% in controls [105]. Such enrichment was not found in non-dopaminergic neurons. Overall, among the 40 patients, 31 (77.5%) had at least one dopaminergic neuron showing a gain of *SNCA* copies as compared to 10/25 (40%) of the controls. These results suggest that late-somatic copy number gain of *SNCA* is not a rare event and suggest that a significant enrichment should be required to trigger the disease. The fact that picomolar concentrations of *SNCA* oligomers can induce disease-related pathways in cells in vitro seems to be in paradox with the latter study [68]. If replicated, these results obtained on nigral neurons would question the hypothesis that low-level mosaics alone would be sufficient to trigger diffuse neurodegenerative disease in vivo. Other brain regions may also be studied as well as other mutations and their putative functional consequences.

In addition to CNVs affecting known disease genes, post-zygotic CNVs may also affect novel Parkinson’s disease genes. In phenotypically discordant monozygotic twin pairs with one of the twins exhibiting Parkinson’s disease, a few post-zygotic novel CNVs have been identified in the affected twins [27]. Further research is needed to confidently link these genes to Parkinson’s disease.

The presence of brain-specific single nucleotide mutations has been assessed in 1988—before the identification of the first causative genes of autosomal-dominant EOAD. In an exploratory study, the sequence encoding the Aβ peptide was analyzed in cDNA isolated from three brains with sporadic AD but no mutation was found [180]. The hypothesis that post-zygotic mutations could explain sporadic AD was reassessed after identification of *APP*, *PSEN1*, and *PSEN2* germline pathogenic mutations in autosomal-dominant families. The analysis of DNA isolated from bulk brain pieces of 99 patients with sporadic AD revealed a *PSEN1* mutation that was eventually confirmed to be present in the germline [139]. This hypothesis was also assessed in Parkinson’s disease and ALS before the era of NGS, with negative results [121, 135]. Recently, WES was performed in hundreds of brains from patients with different types of AOND. While pathogenic mutations were detected in autosomal-dominant genes, parental DNA was not available for testing and the average depth of sequence coverage did not allow for the detection of low-level somatic mutations [76].

The use of deep-sequencing or blood–brain duo strategies has been applied only recently in sporadic AD. In a first study, a targeted deep-sequencing approach was used to analyze the genomic loci of *APP*, *PSEN1*, *PSEN2* and *MAPT* in DNA isolated from the entorhinal cortex of 72 patients with sporadic AD and 58 controls [152]. Custom capture and deep sequencing of the genomic regions of these four genes revealed 107 candidate post-zygotic mutations but only 3 could be confirmed by amplicon-based deep sequencing: two novel *MAPT* missense mutations of unknown significance in sporadic AD patients (variant allele frequencies of 1.0% and 1.1%) and one known *PSEN2* likely benign missense mutation (variant allele frequency of 1.6–5.7%) in a control. Of note, among the 41 patients with an available age at onset, the median age of onset was 78 years (range: 46–92) and among the other 31 other patients, the median age at death was 79 years (range 57–96, the youngest carried a pathogenic *PSEN1* germline variant p.H163R), suggesting that majority had a late disease onset. Another recent study focused on more technical aspects in the context of AD, but did not provide results directly relevant to AD itself [57]. In a study including 17 sporadic AD patients, 2 controls, and 2 patients with vascular dementia, WES (mean depth of coverage: 60.8x) was performed on DNA isolated from blood as well as the hippocampus [122]. This strategy did not allow the identification of low-level mosaics and no putatively pathogenic brain-specific mutation was identified. The average age at death was 86.8 years (range 73–94), suggesting that most of them, if not all, presented a late onset of AD.

With the hypothesis that, similar to germline DNM, post-zygotic, including late-somatic mutations causing sporadic AOND may be associated with early-onset forms, we recently performed a targeted deep-sequencing screen of 11 genes in 445 sporadic AD patients (355 blood samples, 100 brain samples), > 80% of which had an early onset [112]. We used single molecule Molecular Inversion Probes (smMIPs) capture followed by deep sequencing and validation with independent ultra-deep sequencing, allowing for very high sensitivity and specificity. We identified nine post-zygotic mutations with allelic ratios ranging from 0.2% to 10.8%. Two of these mutations were predicted to alter the function of SORL1, which is currently considered as a strong risk factor for EOAD. However, no predicted pathogenic post-zygotic mutations in known autosomal-dominant genes could be identified in this large sample.

Even more recently, 102 genes were screened by targeted capture followed by deep sequencing in 173 samples from 54 human brains [75]. Post-zygotic variants were validated by a technology including the use of UMI. Of them, 20 individuals presented with AD and 20 exhibited Parkinson’s disease or dementia with Lewy bodies. Despite the detection of 62 post-zygotic variants, no putatively pathogenic variant was identified.

The preliminary results of the above-mentioned studies do not immediately point to a significant role for post-zygotic mutations in known disease genes sporadic AOND. This may change with improvements in capture as well as sequencing technologies (including the analysis of single cells which is only just starting and is a tremendously promising field) as well as the analysis of many more brain samples, especially from patients with an early onset of disease and the more accurate detection of mosaic structural variations. Taking together all the positive and negative results obtained from control and diseased brains, the assessment of the somatic variant hypothesis in AOND has opened many novel questions. Among them, there is discussion about the minimum amount of pathological seeds, the timing of occurrence, and the regions where these seeds should appear to be sufficient to trigger neurodegenerative diseases. Experiments in animal models may help researchers to answer some of these questions, combined with the application of ultra-sensitive sequencing of multiple brain regions in AOND patients and controls.

## Conclusions

Pathogenic DNMs, while not easy to identify in adult-onset diseases, clearly play an important role in sporadic AOND. Trio-based exome sequencing of patient–parent blood samples has pointed to numerous germline DNMs in both known disease genes as well as novel candidate genes. Larger sample sizes and functional follow-up studies are essential to validate the role of these candidate genes in AOND. Access to affected brain tissue and more sensitive and specific sequencing approaches will be crucial to investigate further the role of brain-specific mutations in AOND. Challenges lie ahead not only in the identification of these mutations but equally in the clinical interpretation of mutations that are present in only a proportion of all cells present.

In this review, we focused on the most common AOND, but examples of germline or post-zygotic DNM in known Mendelian genes causing other adult-onset neurological disorders have been reported (e.g., [46, 49, 115, 159]).

The in-depth genomic analysis of sporadic AOND is opening many exciting novel research directions, from the identification and characterization of novel disease genes and non-coding regions to the single cell analysis of somatic mutations as well as the analysis of seeding-spreading mechanisms leading to neurodegenerative disorders.

### Electronic supplementary material

Below is the link to the electronic supplementary material.

Supplementary material 1 Supplementary Table 1. Germline and post-zygotic de novo mutations in known genes of AD, Supplementary Table 2. Germline and post-zygotic de novo mutations in known genes of the FTLD–ALS spectrum, Supplementary Table 3. Germline and post-zygotic de novo mutations in known genes of Prion disorders, Supplementary Table 4. Germline and post-zygotic de novo mutations in known genes of alpha-synucleinopathies (XLSX 18 kb)

## Box 1. Overview of the most common adult-onset neurodegenerative disorders and genetics aspects in Mendelian forms

Mendelian inheritance corresponds to monogenic disorders, where, in a given individual, mutations in a single gene (whatever its type, single nucleotide change, copy number variation, causing disease in a dominant or recessive manner) are sufficient to cause disease. In most AONDs, it is expected that monogenic forms represent a small minority of all patients, with an enrichment of monogenic forms observed in the more severe and earlier-onset forms. Because of the adult onset of these disorders, mutations can be passed on to the offspring and therefore monogenic AOND can occur in families. In addition, although monogenic Prion disorders are genetically homogeneous (same gene affected in every patient: *PRNP*), monogenic forms of Alzheimer’s disease, frontotemporal lobar degeneration, amyotrophic lateral sclerosis and Parkinson’s disease are genetically heterogeneous: a single mutation in a given gene may be sufficient to cause disease in a given patient, but different genes are mutated in different patients.

### Alzheimer’s disease

Alzheimer’s disease (AD) is the leading cause of dementia. It is characterized by a progressive decline of memory and other cognitive functions related to the spreading of neuronal lesions called neurofibrillary tangles which are mainly composed of hyper- and abnormally phosphorylated Tau protein. These lesions are however not specific to AD. The other mandatory neuropathologic criterion, which is specific to AD, is the presence of senile plaques, the core of which is composed of aggregated Aβ peptide.

AD is a complex disorder in the vast majority of cases. It is commonly postulated that less than 1% of the patients exhibit an autosomal-dominant form, but this may be actually much less. Three genes have been validated as causing autosomal-dominant AD when carrying pathogenic variants: *APP*, *PSEN1*, and *PSEN2* [36, 143, 160, 161]. *APP* encodes the precursor of the Aβ peptide, which results from the sequential cleavage of APP by the β-secretase and the γ-secretase (*PSEN1* or *PSEN2* encoding the catalytic subunits). Pathogenic variants are responsible for the increased production of the Aβ peptide or the production of a more aggregation-prone form. Majority of the pathogenic variants are highly penetrant and result in a disease onset before the age of 65 years (for review, see [113]). Mutation detection rates highly correlate with the combination of ages of onset and family history (Fig. 2).

### Frontotemporal lobar degeneration (FTLD) spectrum

Frontotemporal lobar degeneration (FTLD) is a neurodegenerative disorder affecting mainly the frontal and temporal lobes. It is the second most common cause of dementia before the age of 65, after Alzheimer’s disease [118]. It is characterized by a clinical heterogeneity which is classified into different syndromes. Behavioral frontotemporal dementia (bvFTD) is the most common clinical syndrome. Patients exhibit progressive deterioration of personality, social behavior, and cognitive impairment [138]. Other patients may exhibit a primary language impairment and fulfill the criteria of primary progressive aphasia, itself divided into subtypes. FTLD is better classified based on neuropathologic findings. FTLD-Tau is characterized by neuronal inclusions of the Tau protein. The other subtypes show immunomarquages negative for Tau and positive for ubiquitin. Among the latter ones, the FTLD-TDP subtype is characterized by inclusions of the TDP-43 protein, while the FTLD-FET is positive for the FET family of proteins including the FUS protein. FTLD itself belongs to a spectrum of diseases with amyotrophic lateral sclerosis (ALS) being the main co-occurring phenotype. ALS is a devastating neurodegenerative disorder affecting the primary and secondary motor neurons and typically leading to death 3–5 years following the diagnosis [168]. Some of the patients exhibit ALS, FTLD, or both. Additional phenotypes can also be encountered.

Most of the Mendelian forms of this spectrum are transmitted as an autosomal-dominant trait. Pathogenic variants in the *MAPT* gene encoding the Tau protein cause FTLD-Tau with or without parkinsonism or a related disorder called progressive supranuclear palsy. FTLD-TDP can be caused by rare mutations in *TARDBP* encoding TDP-43 itself (however mainly causing ALS), but it is more commonly associated with hexanucleotide expansions in a non-coding region of *C9ORF72* or protein-truncating variants in *GRN* (for review, see [133]). Mutations in *VCP*, *HNRNPA1* and *HNRNPA2B1* cause an even larger group of disorders adding Paget disease of bone and inclusion body myopathy to the FTLD–ALS spectrum. Mutations in *FUS* have been reported in families with ALS. *SOD1* mutations cause a specific subtype of ALS without FTLD. Other genes have been identified and account for a smaller proportion of patients, each (for review, see [133]).

In the FTLD spectrum, physicians face the difficulty to select patients for genetic screening. Although younger cases and multiplex families are easily recognized as good candidates for genetic studies, there is no clear age or family history-related criteria to help clinicians decide. Different neurodegenerative disorders must be taken into account when considering the presence or absence of a family history. Up to 43% of the FTLD patients presented a first-degree relative with dementia, ALS, or Parkinson’s disease [133]. The reduced penetrance of several mutations including the most common ones (*C9ORF72*, *GRN*) [108] has led some authors to propose genetic analysis to all patients with a phenotype belonging to this spectrum [11, 17, 170]. Given this incomplete penetrance, it is not surprising to find reports of sporadic cases carrying a pathogenic mutation, however, the absence of parental DNA often precluded the assessment of their inheritance (e.g., *GRN* [90], *MAPT* [142]).

### Prion disorders

Prion diseases are a group of neurodegenerative disorders related to the misfolding of the Prion protein encoded by the *PRNP* gene. They are usually classified as familial, “sporadic” or transmissible. The familial forms (~ 15% of the cases) actually mean autosomal dominant and are related to pathogenic mutations in the *PRNP* gene. It is postulated that autosomal-dominant Prion disease is genetically homogeneous, i.e., all cases should carry a pathogenic *PRNP* mutation. The term sporadic is often used to characterize complex forms (all non-autosomal dominant and non-transmissible situations) whatever the presence or absence of any family history. Here we use the term “complex” instead of sporadic and keep “sporadic” for designating patients with a negative family history. The acquired (or transmissible) forms represent a small minority of the cases. Three main clinical syndromes have been described to be associated with *PRNP* pathogenic mutations: Creutzfeldt–Jakob disease (CJD), Gerstmann–Sträussler–Scheinker (GSS) syndrome, and fatal familial insomnia (FFI).

### Parkinson’s disease, synucleinopathies

Alpha-synucleinopathies regroup different neurodegenerative disorders characterized by the aggregation of the α-synuclein protein in the so-called Lewy bodies in the brains. Parkinson’s disease (PD) is a common complex disorder. The typical clinical presentation is an extrapyramidal syndrome characterized by L-Dopa responsive asymmetric resting tremor, akinesia, and rigidity. Additional neurological manifestations and atypical disease courses can be observed and some patients might exhibit dementia in the late stages of the disease. The clinical evolution is thought to be related to the spreading of neuronal lesions from the substantia nigra to other brain areas including the cortex. A minority of the patients present a Mendelian form, either with an autosomal-dominant transmission such as for *SNCA*, the gene-encoding α-synuclein itself, *LRRK2*, or *VPS35* genes, or with an autosomal recessive transmission such as for *PARK2* (Parkin), *PARK7* (DJ-1), or *PINK1* genes, amongst others (for review, see [62]). Dementia with Lewy bodies (LBD) is the second most common neurodegenerative cause of dementia in the elderly after AD. It shares clinical and pathological features with Parkinson’s and Alzheimer’s diseases and is associated with widespread Lewy bodies in the brain including cortical areas. There is little evidence of families with an autosomal-dominant transmission. Mutations in genes known to cause Mendelian forms of other AOND have been identified in LBD patients, including mutations or triplications of *SNCA*. However, little is still known about LBD genetics (for review, see [176]) and the co-existence of Lewy bodies with AD pathology in some patients can be confusing, including some cases with pathogenic mutations in AD genes (*APP* duplications, *PSEN1/2* variants [59, 74, 176]).

Multiple system atrophy (MSA) is another α-synucleinopathy. The main clinical characteristics include an extrapyramidal syndrome, a cerebellar syndrome, and autonomic dysfunction. In MSA, the α-synuclein protein aggregates primarily in oligodendrocytes, forming glial cytoplasmic inclusions. The genetics of MSA are debated. It is postulated that it is a common complex disorder. Recently, bi-allelic mutations in the *COQ2* gene have been identified in multiplex families with MSA, but the role of this gene is debated due to negative replication studies [72, 104, 107, 119, 137, 156, 158]. There is no evidence of autosomal-dominant MSA and of DNM in this disorder to date (for review see [89]).
